# Properties and fate of human mesenchymal stem cells upon miRNA let-7f-promoted recruitment to atherosclerotic plaques

**DOI:** 10.1093/cvr/cvac022

**Published:** 2022-03-03

**Authors:** Virginia Egea, Remco Theodorus Adrianus Megens, Donato Santovito, Sarawuth Wantha, Richard Brandl, Wolfgang Siess, Sajjad Khani, Oliver Soehnlein, Alexander Bartelt, Christian Weber, Christian Ries

**Affiliations:** Institute for Cardiovascular Prevention (IPEK), Ludwig-Maximilians-University of Munich, Munich, Germany; Institute for Cardiovascular Prevention (IPEK), Ludwig-Maximilians-University of Munich, Munich, Germany; German Center for Cardiovascular Research (DZHK), Partner Site Munich Heart Alliance, Munich, Germany; Department of Biomedical Engineering, Cardiovascular Research Institute Maastricht, University of Maastricht, Maastricht, The Netherlands; Institute for Cardiovascular Prevention (IPEK), Ludwig-Maximilians-University of Munich, Munich, Germany; German Center for Cardiovascular Research (DZHK), Partner Site Munich Heart Alliance, Munich, Germany; Institute for Genetic and Biomedical Research (IRGB), UoS of Milan, National Research Council (CNR), Milan, Italy; Institute for Cardiovascular Prevention (IPEK), Ludwig-Maximilians-University of Munich, Munich, Germany; St. Mary’s Square Institute for Vascular Surgery and Phlebology, Munich, Germany; Institute for Cardiovascular Prevention (IPEK), Ludwig-Maximilians-University of Munich, Munich, Germany; Institute for Cardiovascular Prevention (IPEK), Ludwig-Maximilians-University of Munich, Munich, Germany; Institute for Cardiovascular Prevention (IPEK), Ludwig-Maximilians-University of Munich, Munich, Germany; Department of Physiology and Pharmacology (FyFa), Karolinska Institutet, Stockholm, Sweden; Institute for Experimental Pathology (ExPat), Center for Molecular Biology of Inflammation (ZMBE), Westfaelische Wilhelms-University of Muenster, Muenster, Germany; Institute for Cardiovascular Prevention (IPEK), Ludwig-Maximilians-University of Munich, Munich, Germany; German Center for Cardiovascular Research (DZHK), Partner Site Munich Heart Alliance, Munich, Germany; Institute for Diabetes and Cancer (IDC), Helmholtz Center Munich, Neuherberg, Germany; Department of Molecular Metabolism, Sabri Ülker Center for Metabolic Research, Harvard T.H. Chan School of Public Health, 665 Huntington Avenue, Boston, MA 02115, USA; Institute for Cardiovascular Prevention (IPEK), Ludwig-Maximilians-University of Munich, Munich, Germany; Department of Biochemistry, Cardiovascular Research Institute Maastricht, University of Maastricht, Maastricht, The Netherlands; Munich Cluster for Systems Neurology (SyNergy), Munich, Germany; Institute for Cardiovascular Prevention (IPEK), Ludwig-Maximilians-University of Munich, Munich, Germany

**Keywords:** MSC, Chemotaxis, MicroRNA, Atheroma, MMPs

## Abstract

**Aims:**

Atherosclerosis is a chronic inflammatory disease of the arteries leading to the formation of atheromatous plaques. Human mesenchymal stem cells (hMSCs) are recruited from the circulation into plaques where in response to their environment they adopt a phenotype with immunomodulatory properties. However, the mechanisms underlying hMSC function in these processes are unclear. Recently, we described that miRNA let-7f controls hMSC invasion guided by inflammatory cytokines and chemokines. Here, we investigated the role of let-7f in hMSC tropism to human atheromas and the effects of the plaque microenvironment on cell fate and release of soluble factors.

**Methods and results:**

Incubation of hMSCs with LL-37, an antimicrobial peptide abundantly found in plaques, increased biosynthesis of let-7f and N-formyl peptide receptor 2 (FPR2), enabling chemotactic invasion of the cells towards LL-37, as determined by qRT-PCR, flow cytometry, and cell invasion assay analysis. In an *Apoe*^−/−^ mouse model of atherosclerosis, circulating hMSCs preferentially adhered to athero-prone endothelium. This property was facilitated by elevated levels of let-7f in the hMSCs, as assayed by *ex vivo* artery perfusion and two-photon laser scanning microscopy. Exposure of hMSCs to homogenized human atheromatous plaque material considerably induced the production of various cytokines, chemokines, matrix metalloproteinases, and tissue inhibitors of metalloproteinases, as studied by PCR array and western blot analysis. Moreover, exposure to human plaque extracts elicited differentiation of hMSCs into cells of the myogenic lineage, suggesting a potentially plaque-stabilizing effect.

**Conclusions:**

Our findings indicate that let-7f promotes hMSC tropism towards atheromas through the LL-37/FPR2 axis and demonstrate that hMSCs upon contact with human plaque environment develop a potentially athero-protective signature impacting the pathophysiology of atherosclerosis.

## Graphical Abstract

**Graphical Abstract:**
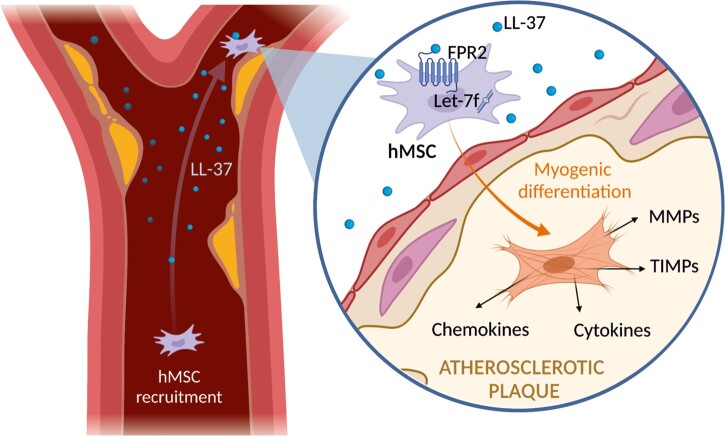

## 1. Introduction

Atherosclerosis represents a chronic inflammatory reaction of the vascular wall in response to dyslipidemia.^1^ Inflammation plays a crucial role in every stage of atherosclerosis from initial onset of the plaque to rupture.^2^ Endothelial dysfunction creates a disbalance between proinflammatory and atheroprotective pathways accompanied by subintimal accrual of atherogenic lipoproteins that triggers the release of various chemotactic factors to initiate and perpetrate leukocyte recruitment, thereby promoting the formation of atherosclerotic plaques.^3,4^ Infiltrating neutrophil granulocytes are thought to drive atherogenesis and plaque destabilization by multiple activities including the release of inflammatory mediators, including LL-37,^5^ which is an antimicrobial peptide consisting of 37 amino acids.^6^ By binding to its cell surface receptors such as N-formyl peptide receptor (FPR)-2, LL-37 exhibits various chemotactic and immunomodulatory functions.^7^ In atherosclerotic plaques, high amounts of LL-37 recruit inflammatory cells and thereby foster atherogenesis.^8,9^

Human mesenchymal stem cells (hMSCs) are found in almost all tissues and are derived from a variety of different sources, including bone marrow, adipose tissue, and peripheral blood.^10^ hMSCs carry the potential to differentiate into multiple cell types of the mesodermal and myogenic lineages, which is determined by environmental stimuli.^11^ Furthermore, in response to their environment, hMSCs secrete a broad range of chemokines, cytokines, and growth factors, conferring immunomodulatory and anti-fibrotic properties to hMSCs.^12^ These features combined with their pronounced tropism to sites of injury and inflammation make hMSCs an attractive therapeutic target.^12–14^ In atherosclerosis, hMSCs are thought to rather have a protective role,^15^ but the underlying cellular and molecular mechanisms are largely unclear.

The family of matrix metalloproteinases (MMPs) regulate tissue remodelling by degrading components of the extracellular matrix.^16^ Especially MMP-2, MMP-9, and MMP-14 enable migration of immune cells, tumour cells, and stem cells through matrix barriers such as basement membranes. In addition, MMPs possess regulatory functions by proteolytically processing cytokines, growth factors, and their receptors.^17^ The activity of MMPs is balanced by four specific tissue inhibitors of metalloproteinases (TIMPs).^16^ Independent of their MMP inhibitory properties, TIMPs also act like cytokines controlling processes such as cell growth, apoptosis, and differentiation.^18^ Their multifaceted roles implicate MMPs and TIMPs in numerous normal and pathophysiological processes.^19^ In atherosclerosis, MMPs have ambivalent roles during late-stage progression and rupture of plaques.^20,21^

Post-transcriptional control of gene expression by microRNAs (miRNAs) is an important regulatory mechanism involved in most biological processes, including stem cell function and human disease such as atherosclerosis.^22–24^ miRNAs are non-coding RNAs capable of gene silencing. This effect occurs by antisense binding to specific target mRNAs resulting in degradation or translational repression of the transcripts. The *lethal-7* (*let-7*) gene was discovered as one of the first miRNAs and is highly conserved across animal species, reflecting its crucial role in development and stem cell biology.^25^ Meanwhile, the let-7 family comprises 12 members known to play important roles in development.^26^ Accordingly, let-7 is typically expressed at high levels in differentiated cell types but is almost undetectable in embryonic stem cells.^27^ Our own data have revealed that accumulation of let-7f in hMSCs is associated with an activated cellular status, improved chemotactic invasion towards inflammatory cytokines, and anti-tumour effects.^28,29^

Here, we report on the role of let-7f in hMSC invasion towards LL-37, let-7f-promoted endothelial adhesion of circulating hMSCs at sites of atheromas, and the effect of human plaque specimens on MMP/TIMP secretion and hMSC fate.

## 2. Methods

### 2.1 Cultivation and treatment of cells

hMSCs isolated from bone marrow of healthy persons under informed consent were purchased from Lonza (Cologne, Germany). Each hMSC lot used in the study was tested by Lonza for purity and their ability to differentiate into the osteogenic, chondrogenic, and adipogenic lineage. The cells were positive for CD29, CD44, CD105, and CD166, and negative for CD14, CD34, and CD45. hMSCs were cultured as described previously,^28^ using the StemMACS expansion media with a weekly medium change (Miltenyi Biotec, Bergisch Gladbach, Germany). All studies were carried out with hMSCs between the fifth and seventh passage of cultivation which exhibited an average cell doubling time of 96 h. For experiments under serum-free conditions, hMSCs were washed with serum-free medium and incubated in Dulbecco Modified Eagle Medium (DMEM) (PAA Laboratories, Coelbe, Germany) supplemented with 1% Nutridoma SP (Roche Applied Science, Mannheim, Germany) in the absence or presence of LL-37 (Anaspec, Fremont, CA, USA) at physiological concentrations of 10–100 ng/mL LL-37 as determined in human blood plasma.^30^ Cells were routinely tested for mycoplasma contamination. Cell viability was determined by trypan blue staining and periodically by application of the WST-8 assay (Dojindo, Rockville, MD, USA).

### 2.2 qRT-PCR analysis of mRNA and miRNA expression

Isolation of total RNA from hMSCs was accomplished using the RNeasy Mini Kit (Qiagen, Hilden, Germany), and on-column DNase digestion with the RNase-free DNase-set (Qiagen) was performed according to the manufacturer’s protocols. The cDNA synthesis was completed following the instructions of the First Strand cDNA Synthesis Kit for RT-PCR (AMV) (Roche Applied Science) using oligo dT primers.

qRT-PCR was carried out on a LightCycler (Roche Applied Science) using LightCycler-FastStart DNA Master SYBR Green I Kit [Roche Applied Science glyceraldehyde-3-phosphate dehydrogenase (GAPDH) as a housekeeping gene standard] according to a previously published protocol.^31^ PCR primer sets and kits were applied as listed in Supplementary material online, *Table S1*.

miRNA expression was determined using the miScript PCR System (Qiagen) for conversion of RNA into cDNA and SYBR Green-based qRT-PCR for detection. Relative expression was normalized to the mean threshold cycle (C_T_) values of multiple reference genes according to a previously published method,^32^ namely snord61, snord72, or U2 that were selected for their low variability in C_T_ values during hMSC treatment. For assessment of let-7f copy numbers, a standard curve was constructed with serial dilution of cDNA synthetized from a known concentration of synthetic let-7f. The respective primer sequences and catalogue numbers are provided in Supplementary material online, *Table S1*.

### 2.3 PCR array analysis

To investigate hMSCs for tissue extract-induced changes in the expression of 84 genes which are potentially relevant in the pathophysiology of atherosclerotic lesions, we applied the Human Cytokines & Chemokines RT^2^ Profiler PCR Array (PAHS-150Z) (Qiagen) by exactly following the protocol provided by the manufacturer. Briefly, 300 ng of mRNA was isolated from hMSCs and reverse-transcribed to cDNA, mixed with a ready-to-use PCR master mix, and aliquoted in equal volumes across a 96-well array plate. The samples were then subjected to RT-PCR performed on a LightCycler 96 device (Roche Applied Science). The expressions of target genes were measured relative to the mean threshold cycle (C_T_) values of 5 housekeeping genes included on the plate according to a previously published method.^32^ Data analysis was performed according to the ΔΔC_T_ method with normalization of the raw data to the housekeeping genes using a web-based software application provided by the array manufacturer (Qiagen). The results were expressed as the relative fold regulation of gene expression in two groups as indicated. Genes with relative fold changes of at least ±2 were considered as up- or down-regulated in expression.

### 2.4 Transfection of cells with let-7f mimics

To study miRNA function in hMSCs, synthetic let-7f-5p (let-7f mimics) and non-specific siRNA control oligonucleotides were applied (Qiagen). The sequences of mimics and control are listed in Supplementary material online, *Table S1*. Cells were transfected with 20 nM of miRNA or siRNA by use of Lipofectamin 2000 (Invitrogen) as described previously.^28^

### 2.5 Protein extraction and western blot analysis

Whole protein extraction from hMSCs was accomplished with a buffer containing 40 mM Tris–HCl pH 8.0, 150 mM NaCl, 1% NP-40, 0.5% sodium deoxycholate, and 0.1% SDS supplemented with a mixture of proteinase and phosphatase inhibitors (cOmplete Mini Tablets and PhosSTOP; Roche).

Fifteen micrograms of protein per lysate were resolved by SDS/polyacrylamide gel electrophoresis and then transferred to polyvinylidene difluoride membranes as described.^31^ The blotted membranes were incubated overnight with the primary antibodies enlisted in Supplementary material online, *Table S2* and then with HRP-conjugated secondary antibodies (Cell Signaling Technology). Bound antibodies were detected using the enhanced chemiluminescence system (GE Healthcare Life Sciences, Freiburg, Germany). Recombinant protein standards (Invitrogen, Karlsruhe, Germany) were used for molecular mass determination. Densitometric quantification was performed using a GS-800 Calibrated Densitometer driven by ImageMaster-1D Elite quantification software (GE Healthcare Life Sciences) as recommended by the distributor.

### 2.6 Flow cytometry analysis

FPR2 surface expression on hMSCs was assessed by flow cytometry. Cells were incubated with anti-human FPR2 (1:100) in 50 μL of a 1:1 mixture of Hank’s complete and FACS staining buffer. Cells were washed with Hank’s complete and directly analysed by flow cytometry using a FACS Canto II (BD Biosciences, Franklin Lakes, USA). Data were analysed with FlowJo Software (Tree Star Inc, Ashland, USA).

### 2.7 Cell invasion assay

The ability of hMSCs for directed migration through a barrier of human ECM towards a gradient of chemoattractants was analysed using the Costar Transwell chamber system (Costar, Pleasanta, CA, USA) as previously described in detail.^31^ Briefly, membrane filters with a pore size of 8 μm (Costar) were coated with 10 μg human ECM (BD Biosciences, Bedford, MA, USA) which is mainly composed of laminin, collagen type IV, and proteoglycans, providing a composition similar to that of human basement membranes. hMSCs (5 × 10^3^) were placed into the upper compartment of the invasion chamber. The lower compartment of the Transwell system contained LL-37 as chemoattractant or serum-free medium as control. Each invasion experiment was performed in triplicate. After 48 h of incubation, cells that had migrated into the lower compartment were counted. The invasion rate was calculated from the number of migrated cells to the total cell number.

For inhibition experiments, hMSCs were pre-incubated for 30 min without or with 100 μM tert-butyl-oxycarbonyl-Phe-Leu-Phe-Leu-Phe (BOC-PLPLP) (Tocris, Bristol, Great Britain), a specific antagonist of LL-37 binding to FPR2.^33^ Thereafter, the cells were transferred into the upper compartment of the Transwell system. The inhibitor was also added at the same concentrations to the medium in the upper and lower compartment. Preceding measurements had shown that incubation of hMSCs with BOC-PLPLP at 100 nM for 48 h did not significantly affect cell viability and proliferation.

### 2.8 Immunocytochemistry analysis

To examine the expression of αSMA and caldesmon in hMSCs, we deployed immunocytochemistry analysis as previously described.^34^ hMSCs were seeded in glass coverslip bottom chamber 8-well slides (Ibidi, Planegg, Germany) before experimental treatments. Upon completion of treatments, cells were fixed with 4% paraformaldehyde (PFA) for 15 min and permeabilized with 0.1% Triton X-100 in PBS/BSA 1% for 30 min at room temperature. The slides were subsequently incubated overnight at 4°C with primary antibody (Supplementary material online, *Table S2*). Non-specific isotype antibodies were used as negative controls. Species-specific fluorescently conjugated secondary antibodies were applied for 2 h at room temperature (Supplementary material online, *Table S2*). The slides were embedded in Prolong^®^ Diamond antifade mountant (ThermoFisher Scientific) with added 4′,6-diamidino-2-phenylindole (DAPI) to counterstain nuclei. Analysis was performed at 20°C using the Olympus IX70 microscope (Olympus Europa GmbH, Hamburg, Germany) with objective lens Olympus × 20 (numerical aperture 0.55), and images were taken using SensiCam camera (PCO Imaging, Kelheim, Germany) with Image-ProPlus software (Media Cybernetics, Bethesda, MD, USA).

### 2.9 Animal experiments and intravital fluorescence microscopy

*In vivo* mouse experiments were performed to analyse hMSC adhesion at the endothelium of the aortic root. *Apoe*^–/–^ mice on C57Bl/6J background were fed with high-fat diet (21% fat, 0.15% cholesterol) for 4 weeks to induce the formation of atheromatous tissues (plaques) at the area of the aortic root. Prior to the experiments, hMSCs were subjected to live cell-labelling applying a membrane-permeable green fluorescent dye, calcein-AM (Invitrogen). Adhesion of the cells to the endothelium at atheromatous tissue sites of the carotid artery biforcation was then analysed by intravital microscopy as previously reported^35^ with some modifications. In brief, mice (*n* = 3) were placed in a supine position, and the right jugular vein was cannulated with a catheter for injection of 10^6^ calcein-labelled hMSCs per animal. The cells were allowed to circulate for 5 min. With the use of an Olympus BX51 microscope equipped with a Hamamatsu 9100-02 EMCCD camera and a 10× saline-immersion objective, pictures of the carotid artery biforcation section were acquired and analysed. Green fluorescent protein–expressing cells were considered hMSCs. Adherent cells were defined as those remaining stationary for ≤30 s. The animals were anesthetized once, i.p., with 90 mg/kg ketamine and 10 mg/kg xylazine. Killing of animals was achieved by cervical dislocation under deep anaesthesia. All animal experiments were approved by the local ethics committee (permission number ROB 55.2-1-542532-159-11) and performed in accordance with institutional guidelines.

### 2.10 *Ex vivo* artery perfusion and two-photon laser scanning microscopy

Carotid arteries of C57Bl/6J wildtype mice or *Apoe*^–/–^ fed with high-fat diet for 2 weeks were carefully explanted, mounted in a customized perfusion chamber, pressurized at 80 mm Hg, and incubated with eFluor 450-coupled CD31 antibody (5 µg/mL, 30 min) as described.^36^ Prior to the perfusion experiments, hMSCs transfected with let-7f mimics and non-specific control oligonucleotides were stained with CellTracker Red or Green (Thermo Fischer Scientific), respectively, and washed carefully to avoid contamination of fluorescence signal in the arterial wall. In order to perform a comparative *in situ* analysis of hMSCs featuring high or low levels of let-7f expression on the ability of these cells to adhere at arterial endothelium, a 1:1 mixture of red and green labelled hMSCs (10^6^ cells in total) were perfused for 10 min through the mounted and pressurized vessel at 0.54 mL/min after which the vessel was flushed with HBSS in order to wash out non-adherent cells. Adherent hMSCs were visualized using the Leica SP5 II MP two-photon laser scanning microscopy (Leica Microsystems, Wetzlar, Germany) as described^37^ while the artery remained pressurized (80 mmHg). The total numbers of adherent hMSCs and controls were counted per surface area unit. Numbers of adherent hMSCs were determined in 8 randomly selected areas by two independent observers.

For 3D image acquisition post-perfusion, *z*-stacks of the mounted carotid arteries were visualized using the two-photon laser scanning microscope with a pre-chirped and pulsed Ti: Sapphire Laser (Spectra Physics, MaiTai Deepsee, Darmstadt, Germany) tuned at 800 nm and a 20 × NA1.00 (Leica) water dipping objective. Spectral detection was performed utilizing Hybrid Diode detectors (*n* = 4) tuned for maximum intensity of the (auto)fluorescence signal. Image acquisition (second harmonics generation of collagen and autofluorescence; 390–470 nm, cell tracker green + autofluorescence; 480–550 nm, cell tracker red: 600–640 nm). Image processing was performed using LASX software including 3D analysis plugin (Leica).

### 2.11 Isolation of human carotid atherosclerotic plaque material and incubation with hMSCs

Atherosclerotic plaques were obtained from patients who underwent endarterectomy for high-grade stenosis of the internal carotid artery. Patient informed consent was obtained as approved by the Ethics Committee of the Faculty of Medicine of the University of Munich and in accordance with the ethical principles for medical research involving human subjects as set out in the Declaration of Helsinki. The carotid plaque tissue was endarterectomized, processed, and preserved as described^38,39^ with some modifications (Supplementary material online, *Figure S1*). Briefly, plaque homogenates from 5 patients, each 50 mg wet weight per mL buffer containing 150 mM NaCl (Sigma) and 1 mM EDTA (Sigma) pH 7.4, were mixed to obtain pooled plaque sample. From another patient, atherosclerotic plaque material as well as adjacent normal intima was preserved by vascular surgery of a stenotic femoral artery. The crude tissue homogenates were vortexed for 1 min, subjected to sonification (Branson Sonic Power Company, Danbury, CT, USA) for 1 min, kept on ice for 10 min, and then centrifuged (20 000 *g*, 20 min, 4°C). The supernatant fractions of pooled plaque extracts and the plaque extract with corresponding intima extract were retained and stored in aliquots at −80°C.

For incubation with hMSCs, the extracts were thawn, centrifuged, mixed with serum-free medium at a final dilution of 1:100, and then added to subconfluent cell cultures. After 24 h, cells were lysed for mRNA isolation. For analysis of hMSC associated and secreted proteins, the cells were cultivated with and without 1:100 diluted tissue extracts for 6 days. Then, the conditioned medium was removed from confluent cultures, the cells were washed, and incubated in serum-free medium for 24 h. Subsequently, conditioned media were collected and the proteins extracted from the cells and stored at −20°C as described.^40^

### 2.12 Statistical, gene ontology, and pathway analysis

Statistical analysis was performed using Prism 8.0 (GraphPad Software, La Jolla, CA, USA). Unless otherwise noted, descriptive statistics included mean and standard deviation or 95% confidence intervals. Data distribution and homogeneity of variance were tested by the Shapiro–Wilk and Levene’s test, respectively. Comparisons between two groups were performed by Student’s *t*-test, with Welch correction when appropriate. Comparisons among >2 groups by one- or two-way ANOVA with Tukey or Bonferroni *post hoc* test or Kruskal–Wallis test with Dunn *post hoc* test, as indicated. Differences in the adhesion between let-7f and negative control-siRNA transfected hMSCs were assessed by using Wilcoxon test on ratios of each field of view to avoid assumptions of differences in visualized cells per field and not-normal distribution of the data. A two-tailed *P*-value <0.05 was considered as statistically significant. Each experiment was independently repeated ≥3 times. Biological process gene ontology (GO) annotations were used for pathway enrichment analysis. The clusterProfiler version 3.0.4 software package for R was deployed for the analysis and visualization of enriched pathways using the Bonferroni’s correction of *P*-values.

### 2.13 Ethics statements

Animal experiments were approved by the Regierung von Oberbayern and performed in accordance with the guidelines from Directive 2010/63/EU of the European Parliament on the protection of animals used for scientific purposes or the NIH Guide for the Care and Use of Laboratory Animals.

For use of human plaque tissue specimens, patient consent was obtained as approved by the Ethics Committee of the Faculty of Medicine of the University of Munich. The investigations conform to the principles outlined in the Declaration of Helsinki.

## 3. Results

### 3.1 LL-37 attracts hMSCs by up-regulating let-7f- and FPR2-mediated cell invasion

**Figure 1:**
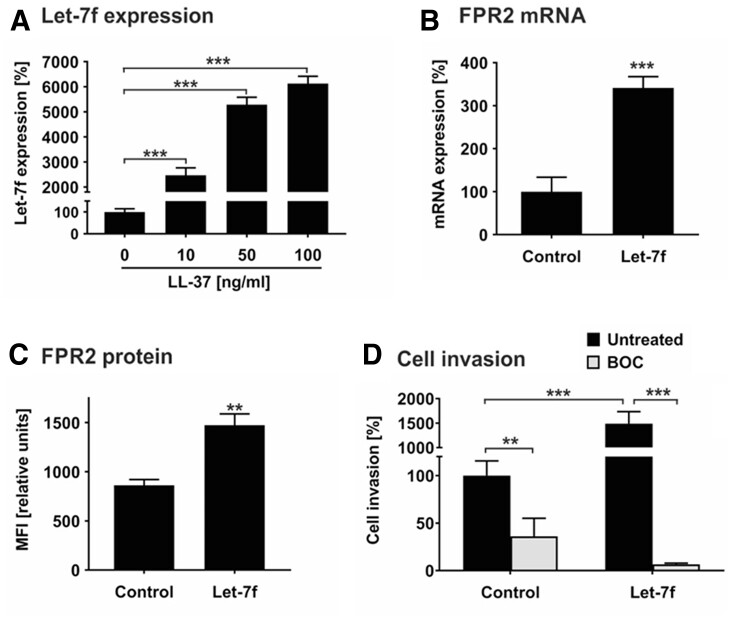
Let-7f promotes LL-37-directed invasion of hMSCs. (*A*) hMSCs were incubated with 0 (set as 100%), 10, 50, and 100 ng/mL LL-37 and cultivated under serum-free conditions. After 24 h, miRNA expression of let-7f was quantified by qRT-PCR. The values shown were normalized to snoRNAs (SNORD61, SNORD72, U2). (*B*) hMSCs transfected with synthetic let-7f miRNA mimic (let-7f) or non-specific oligonucleotides (control, C) were cultivated for 3 days and analysed for FPR2 mRNA expression by qRT-PCR. The values were normalized to GAPDH and are given in percent to control set as 100%. (*C*) hMSCs transfected with let-7f mimics or unspecific control siRNA were incubated for 3 days before analysis. FPR2 protein expression on the cell surface was quantified by flow cytometry analysis. Results are given in mean fluorescence intensity (MFI). (*D*) hMSCs transfected with let-7f mimics or control siRNA were placed into the upper compartment of Transwell cell migration inserts that had been coated with human ECM. The lower compartment contained 100 ng/mL LL-37 as chemoattractant. After 2 days of incubation in the absence and presence of 100 nM BOC-PLPLP (BOC), a LL-37 receptor antagonist, cells that had been migrated into the lower compartment of the Transwell system were quantified. Untreated control cells were set 100%. All results are given as mean values ± SD of triplicate measurements (*n* = 3). **P < 0.01; ***P < 0.001 for treated cells compared to controls as computed by Student’s unpaired *t*-test (*A–C*) and two-way ANOVA with Bonferroni *post hoc* test (*D*).

Inflamed tissues release cytokines and chemokines, which recruit hMSCs in a let-7f-dependent fashion.^29,31^ Atherosclerotic plaques are inflammatory lesions producing elevated levels of LL-37 thought to contribute to the progression of the disease.^4,9^ Therefore, we tested the hypothesis that LL-37 has an effect on let-7f-controlled migration in hMSCs. Incubation of hMSCs with LL-37 caused a significant augmentation of endogenous let-7f miRNA levels in a dose-dependent manner in comparison to untreated cells (*Figure 1A*). Two RNA binding proteins that act as natural inhibitors of let-7f, LIN28A, and LIN28B, were undetectable in untreated or LL-37-treated hMSCs (data not shown). FPR2 is the endogenous cell surface receptor of LL-37. To investigate the influence of let-7f on FPR2 expression in hMSCs, we analysed the effect of increasing let-7f by transfection with synthetic let-7f molecules (mimics) or non-specific oligonucleotides (control). The overexpression of let-7f in hMSCs led to an increase of FPR2 mRNA and protein expression as demonstrated by qRT-PCR and flow cytometry analysis, respectively (*Figure 1B and C*).

Tissue invasion requires cells to migrate through barriers of extracellular matrix. We tested the role of LL-37 and let-7f in this process by applying the Transwell cell invasion assay. In the presence of LL-37 as sole chemoattractant in the lower compartment of the Transwell chamber, hMSC control cells transfected with non-specific oligonucleotides exhibited a pronounced directed invasion rate set as 100% (*Figure 1D*). The overexpression of let-7f in hMSCs by transfection with mimics enhanced the LL-37-directed invasive capabilities of the cells by 15-fold compared to control cells transfected with non-specific oligos (*Figure 1D*). By the addition of BOC-PLPLP, an antagonist for the binding of LL-37 to FPR2,^41^ both basal and let-7f mimic-stimulated invasion of hMSCs towards LL-37 were significantly diminished (*Figure 1D*).

Together, these findings indicate that LL-37 is a chemoattractant in hMSCs, promoting their invasion by up-regulating let-7f and FPR2 in the cells.

### 3.2 Let-7f promotes adhesion of hMSCs to atheromatous endothelium

**Figure 2:**
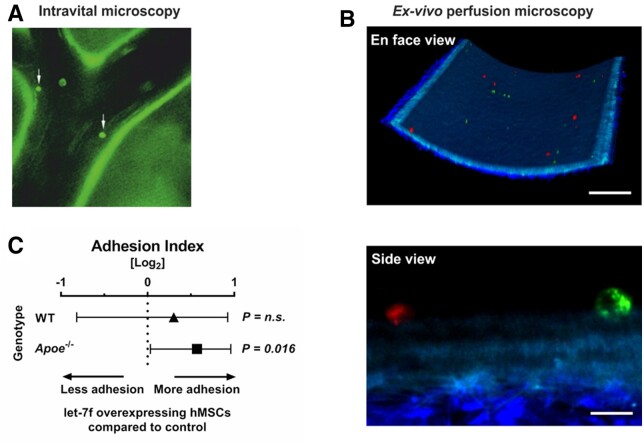
hMSC adhere to murine carotid arteries. (*A*) hMSCs labelled with a cell tracker were adoptively transferred into *Apoe^−/−^* mice fed with high-fat diet for 4 weeks and interactions with the arterial endothelium of the carotid artery was visualized by epifluorescence microscopy (white arrows). (*B*) Carotid arteries were explanted from *Apoe*^–/–^ mice fed with high-fat diet. A 1:1 mixture of red and green labelled hMSCs transfected with let-7f mimics or non-specific oligonucleotide control were perfused through the mounted vessel. Adherent hMSCs were detected in randomly selected areas and analysed using two-photon laser scanning microscopy. Scale bars: 100 µm (en face view) and 10 µm (side view). (*C*) Quantification of the *ex vivo* adhesion assay. The Forest plot shows the adhesion index of let-7f-transfected hMSCs (ratio of adherent let-7f-transfected to control siRNA-transfected hMSCs) perfused in mounted carotid arteries of *Apoe^−/−^* (*n* = 5) and wildtype mice (WT) (*n* = 2). Results were collected in 8 different fields of view per mouse (*n* = 40 in *Apoe*^–/–^ and *n* = 16 in WT mice). *P*-values computed by Wilcoxon signed-rank test (*C*). n.s., not significant.

hMSCs are known for their tropism to sites of inflammation.^12^ Atheromatous tissues accumulate multiple inflammatory factors (including LL-37) which are released into circulation.^4^ Hence, we tested the hypothesis that circulating hMSCs may target at atheroprone sites of the carotid arteries *in vivo*. For this, hMSCs labelled with calcein were injected intravenously in *Apoe*^–/–^ mice fed a high-fat diet for 4 weeks to provoke the development of atherosclerotic plaques. After 5 min, cell adhesion at the carotid artery was monitored using intravital epifluorescence microscopy. In all mice analysed in this study (*n* = 3), we detected hMSCs that had attached to the endothelium at predilection sites of atherosclerosis (i.e. carotid bifurcation) compared with atheroprotected area subjected to high-shear stress (*Figure 2A*). This shows that hMSCs adhere to atherosclerotic lesion sites in large arteries *in vivo*. However, the majority of hMSCs upon injection into the mice were likely trapped in the lung microcirculation allowing only low numbers of cells into the periphery.

To overcome this limitation and to determine the influence of let-7f on the ability of hMSCs for adhesion to the inner vessel wall, we adopted a method based on the *ex vivo* perfusion of mouse carotid arteries. To this end, carotid artery explants were prepared from *Apoe*^–/–^ mice that had been kept on high-fat diet (*n* = 5) and wild-type controls (*n* = 2). In each experiment, hMSCs comprising a 1:1 mixture of cells transfected with let-7f (mimics) or non-specific oligonucleotides (control) differentially stained with fluorescent dyes were perfused through the explanted arteries. hMSCs adhering to the endothelium were detected and analysed by two-photon laser scanning microscopy (*Figure 2B* and Supplementary material online, *Video S1*). The results of our studies demonstrated that hMSCs overexpressing let-7f showed significantly increased adhesion to the endothelium of *Apoe*^–/–^ mice in comparison with control cells (*P* = 0.016). Interestingly, this effect was not significantly detected in wild-type mice fed a chow diet (*Figure 2C*).

These findings indicate that hMSCs circulating in peripheral blood are capable of targeting and adhering at predilection sites of atherosclerosis, a process facilitated by elevated levels of let-7f in the cells.

### 3.3 Atherosclerotic plaque affects the hMSC secretome

**Figure 3:**
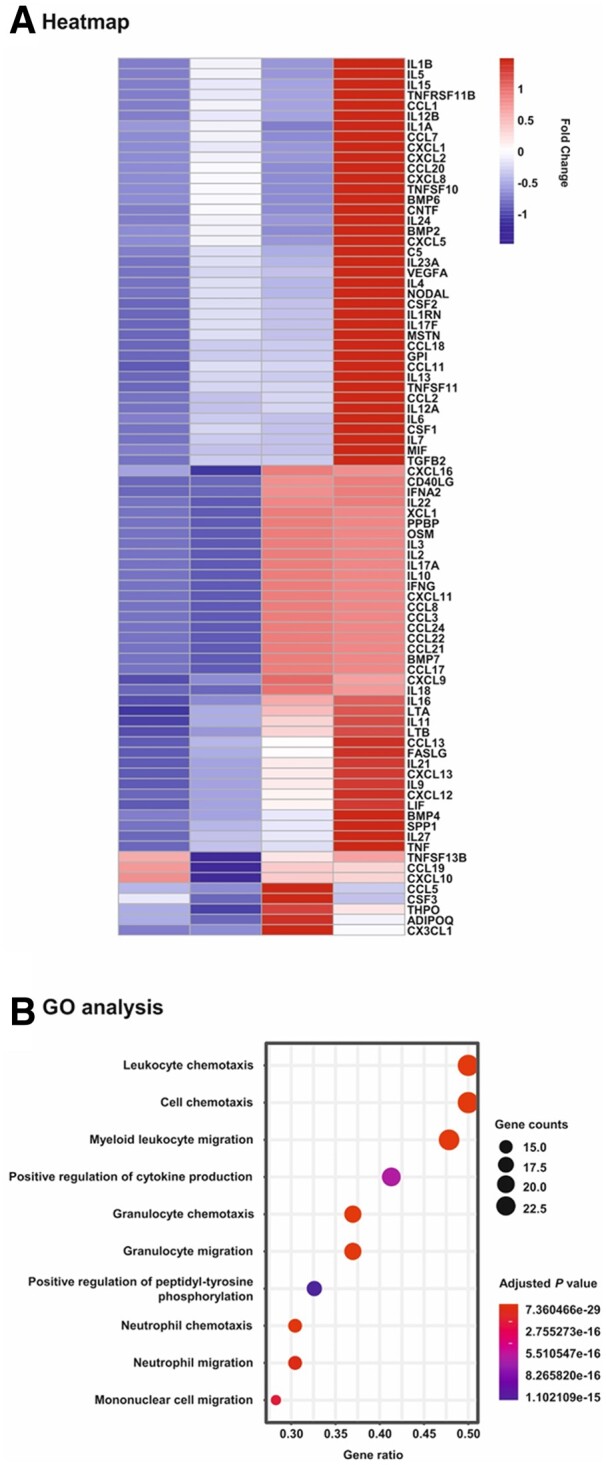
Heatmap and gene ontology analyses of PCR array data on changes in mRNA expression in hMSCs upon exposure to human plaque. hMSCs were cultivated for 24 h in serum-free medium (control) and in the presence of 1:100 dilutions of extracts obtained from human atherosclerotic plaque (Plaque; *n* = 1) and the adjacent normal tissue (Intima; *n* = 1) of a single patient as well as pooled plaques from 5 patients (Pool; *n* = 1). Subsequently, mRNA was isolated and subjected to Human Cytokines & Chemokines RT^2^ Profiler PCR Array analysis. (*A*) Heatmap analysis on the expression levels of all 84 genes analysed in the study. Scale: relative normalized expression. (*B*) Gene ontology (GO) and pathway analysis. The genes were analysed for enrichment in the ontology biological process. The size of the circles is proportional to the number of genes up-regulated more than two-fold within a specified biological process and the colour of the circles reflects the Bonferroni adjusted *P*-value.

To analyse the response of hMSCs to the atherosclerotic plaque microenvironment, specimens from patients who underwent endarterectomy were homogenized and prepared for further use as stimulants (Supplementary material online, *Figure S1*). hMSCs were cultured with diluted plaque homogenates and adjacent normal intima obtained from the same patient as well as in the absence and presence of pooled plaque from five patients. Subsequently, PCR Array technology was performed to determine the transcription of cytokines and chemokines including pro- and anti-inflammatory factors in these cells. As shown in the heatmap (*Figure 3A*), the incubation with plaque lysates affected and mostly up-regulated the transcription of the majority of the 84 genes analysed in this study. Quantitative assessments revealed consistent trends with mostly stronger effects observed for pooled plaque lysates than single patient plaque in comparison to normal intima and untreated cells, respectively (*Table 1*). However, the strongest impact was observed after the incubation with pooled plaque lysates (*Table 1*). Gene ontology (GO) and pathway analysis demonstrated that the exposure of hMSCs to plaque lysates evokes profound alterations in the transcription of genes linked to chemotaxis and migration of cells (*Figure 3B*).

**Table 1:** Genes of PCR array whose expression was up- or down-regulated in hMSCs by at least two-fold following exposure of the cells to plaque

Acc. No.	Symbol	Descriptions	Relative expression^a^	
			Plaque/intima	Pool/control
NM_001200	BMP2	Bone morphogenetic protein 2	2.0	19.3
NM_002986	CCL11	Chemokine (C-C motif) ligand 11; eotaxin	4.0	3.4
NM_005408	CCL13	Chemokine (C-C motif) ligand 13; MCP-4	2.0	2.1
NM_002988	CCL18	Chemokine (C-C motif) ligand 18; PARC	4.7	3.3
NM_000759	CSF3	Colony stimulating factor 3; G-CSF	−2.3	−3.7
NM_002089	CXCL2	Chemokine (C-X-C motif) ligand 2; GRO beta	2.8	11.7
NM_000576	IL1B	Interleukin 1, beta	3.9	12.6
NM_000589	IL4	Interleukin 4	1.8	4.9
NM_000600	IL6	Interleukin 6	1.3	3.6
NM_002188	IL13	Interleukin 13	5.5	3.4
NM_052872	IL17F	Interleukin 17F	6.4	4.5
NM_006850	IL24	Interleukin 24	2.3	16.3
NM_145659	IL27	Interleukin 27	2.2	2.4
NM_005259	MSTN	Myostatin	4.3	4.2
NM_003810	TNFSF10	Tumour necrosis factor (ligand) superfamily, member 10; TRAIL	2.4	68.7

a Genes consistently regulated in both comparison groups: plaque relative to intima (set as 1) and pooled plaques relative to untreated control cells (set as 1).

**Figure 4:**
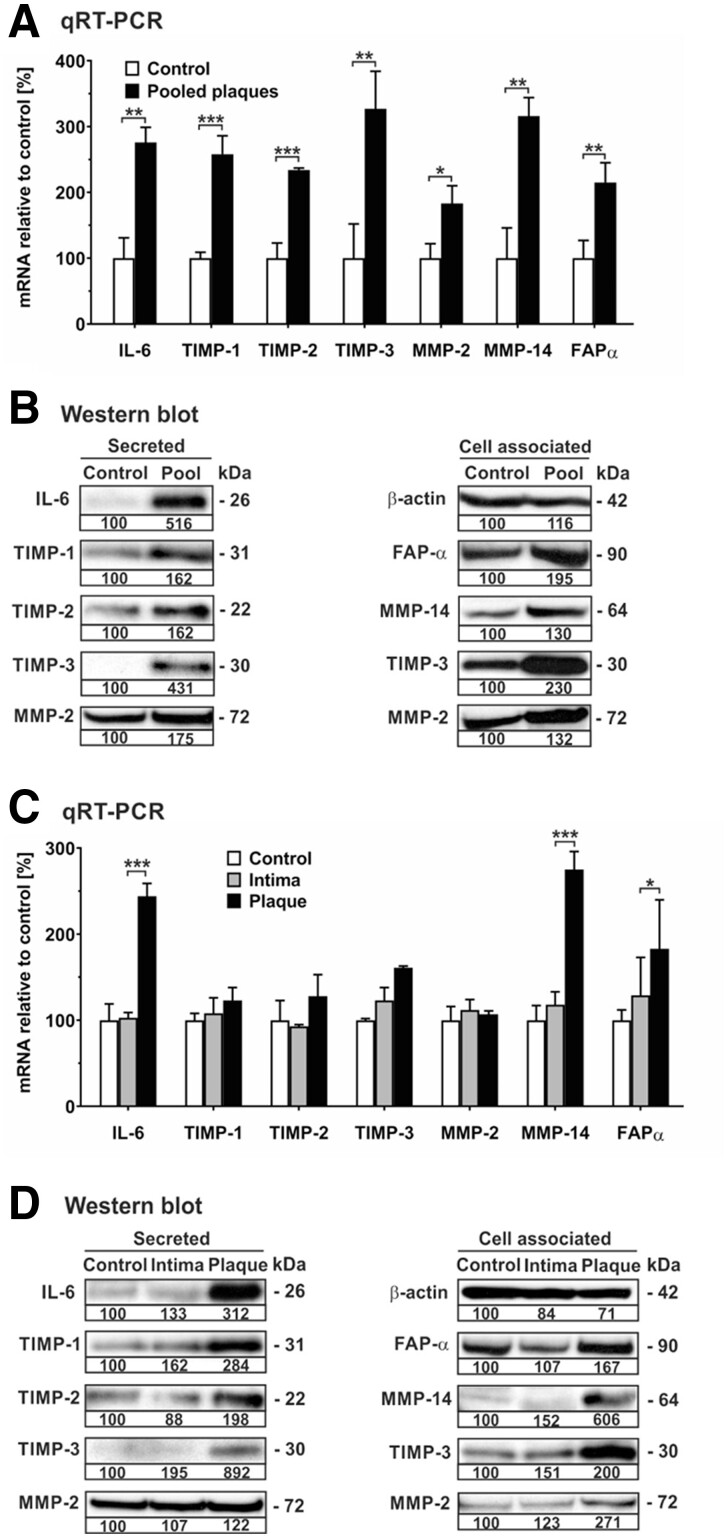
Human plaque increases mRNA expression and release of various proteinases and their inhibitors in hMSCs. hMSCs were cultivated in serum-free medium (control) or in the presence of 1:100 dilutions of pooled protein extracts obtained from human atherosclerotic plaques of five patients (pooled plaque) as well as atherosclerotic plaque (plaque) and adjacent normal intima (intima) from another patient. (*A, C*) After 24 h, cells were analysed for mRNA expression of gene transcripts as indicated using qRT-PCR. The values were normalized to GAPDH and are given in percent to control set as 100%. (*B, D*) Analysis of secreted and cell associated proteins was performed by western blot analysis of aliquots from cell culture supernatants and cell extracts, respectively, of hMSCs after 7 days of cultivation. For densitometric quantification, protein from control cells was set as 100% densitometric units. Intracellular β-actin was detected as loading control. Protein data are representative of three independent experiments (*n* = 3). *P < 0.05, **P < 0.01, ***P < 0.001 for treated cells compared with controls as computed by two-way ANOVA with Bonferroni *post hoc* test (*A, C*).

Chemotactic invasion of hMSCs is facilitated by MMPs, and MMP expression levels in plaque correlate with atherogenesis.^20,31^ Hence, based on the enrichment of chemotaxis and migration pathways in GO analysis, we extended our investigation to include the impact of plaque material on mRNA and protein expression of proteinases and proteinase inhibitors in hMSCs using qRT-PCR and western blot analysis. On the mRNA level, pooled plaque lysates significantly up-regulated the transcription of MMP-2, MMP-14, and fibroblast activation protein α (FAP-α) as well as TIMP-1, TIMP-2, and TIMP-3 in comparison to untreated hMSCs (*Figure 4A*). On the protein level, pooled plaque lysates enhanced the secretion of TIMP-1, TIMP-2, TIMP-3, and MMP-2 as determined by analysing cell culture supernatants (*Figure 4B*). Similarly, pooled plaque lysates augmented levels of proteins in hMSCs known to be associated with the cell surface such as FAP-α, MMP-14, TIMP-3, and MMP-2 in comparison to untreated cells (*Figure 4B*). Very similar effects were obtained when hMSCs were incubated with plaque lysates and adjacent normal intima (*Figure 4C and D*). MMP-9 was undetectable in untreated or treated hMSCs. Furthermore, validation of the mRNA data obtained by the array analysis (*Table 1*) was exemplified by confirmation of plaque-induced transcription and protein secretion of IL-6 in these cells (*Figure 4A–D*).

Taken together, hMSCs respond to soluble factors present in atherosclerotic plaque tissue by increasing production of various cytokines, chemokines, proteinases, and proteinase inhibitors that are potentially influencing plaque stability.

### 3.4 Atherosclerotic plaque induces myogenic differentiation of hMSCs

**Figure 5:**
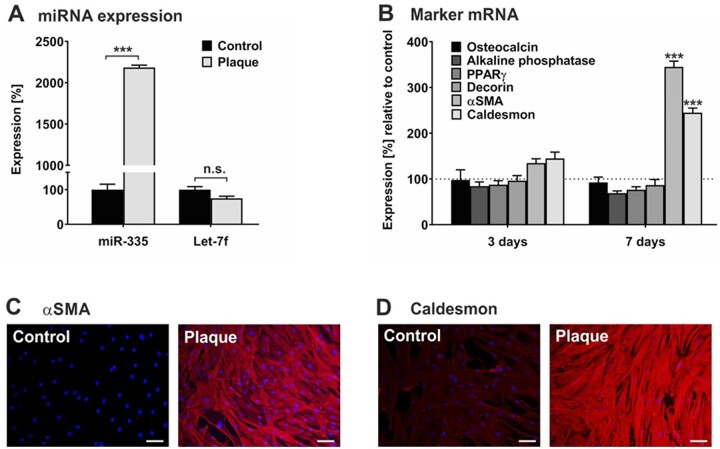
Human plaque promotes myogenic differentiation in hMSCs. hMSCs were cultivated in serum-free medium (control) or in the presence of a 1:100 dilution of pooled protein extracts obtained from human atherosclerotic plaques of 5 patients (plaque). (*A*) After 24 h, RNA was collected and subjected to quantification of miR-335 and let-7f expression levels using qRT-PCR. The values shown were normalized to snoRNA and given in percent to control set as 100%. (*B*) After 3 days and 7 days, cells were analysed for mRNA expression of gene transcripts as indicated using qRT-PCR. The values were normalized to GAPDH and are given in percent to control set as 100%. (*C, D*) After 7 days, cells were analysed by immunocytochemistry for the expression of αSMA (*C*) and caldesmon (*D*) stained with anti-rabbit IgG (Star 635P, red). DAPI (blue) was used for nuclear staining. Magnification ×4. Scale bars, 5 µm. Data shown in *A* and *B* are given as mean values ± SD of triplicate measurements (*n = 3*). Protein data are representative of 3 independent measurements (*n* = 3). n.s., not significant; **P < 0.01, ***P < 0.001 as computed by two-way ANOVA with Bonferroni *post hoc* test (*A, B*).

Depending on the presence of appropriate environmental stimuli, hMSCs are able for differentiation into osteocytic, adipocytic, chondrocytic, or myelogenic cells that are characterized by their transition from a quiescent into an active state.^11,42^ To study the impact of plaque on hMSC fate, we cultured hMSCs in the absence and presence of pooled plaque lysates and first analysed the expression of specific miRNAs associated with hMSC activation. As determined by qRT-PCR analysis, exposure of hMSCs to plaque lysates caused a 20-fold up-regulation of the levels of miR-335, which is known to be up-regulated upon hMSC differentiation,^43^ whilst let-7f transcription remained unchanged (*Figure 5A*). Next, hMSCs incubated in the absence and presence of pooled plaque lysates were examined for mRNA expression of markers of the osteogenic (osteocalcin, ALP), adipogenic (PPARγ), chondrogenic (decorin), and myogenic (αSMA, caldesmon) lineages. Assessment by qRT-PCR revealed a time-dependent increase in mRNA levels of myogenic markers but only minimal change in the transcription rates of osteogenic, adipogenic, and chondrogenic markers determined after 3 and 7 days of cultivation with plaque lysates compared to control (*Figure 5B*). The mRNA findings were confirmed on protein level by immunocytochemistry analysis, demonstrating a pronounced increase of both αSMA and caldesmon in hMSCs upon incubation with plaque lysates in comparison to untreated control cells (*Figure 5C and D*).

These findings suggest that hMSCs recruited into atherosclerotic plaque tissue potentially undergo myogenic differentiation.

## 4. Discussion

Accumulating evidence supports the concept that hMSCs play a protective role during all stages of atherogenesis.^15^ However, their detailed function and action as well as their therapeutic potential remain widely unclear. The aim of our study was to investigate hMSC tropism and response to plaque.

Our study provides evidence that LL-37, a small neutrophil-derived peptide known to be abundant in plasma and plaques of patients with atherosclerosis,^9^ acts as a chemoattractant of hMSCs. The LL-37-directed invasion occurs in a let-7f-dependent way by a mechanism similar to hMSC’s chemotactic invasion towards inflammatory cytokines and chemokines.^29^ This suggests that let-7f is a key regulator in hMSC trafficking to inflamed tissues. LL-37 exerts its effects on cells through FPR2, a common G protein-coupled receptor. FPR2 is predominantly expressed in myeloid cells and elicits pro- and anti-inflammatory responses depending on the cell type or agonist.^44,45^ In hMSCs, LL-37 induces let-7f, which augments FPR2 on the cell surface, suggesting an indirect regulatory role of let-7f by targeting an inhibitor of FPR2 expression in these cells. A similar mechanism of let-7f indirectly up-regulating CXCR4 expression in hMSCs has been previously published by us.^29^ Thus, hMSCs respond to LL-37 by a positive stimulatory loop regulated by let-7f and promoting LL-37/FPR2-mediated chemotaxis towards plaques.

Clinically, erosion and rupture of the atherosclerotic plaque accounts for the majority of fatal acute myocardial infarctions and sudden cardiac deaths.^46,47^ Plaques adopt stable or vulnerable states according to changes of the internal and surrounding environment. For example, large numbers of infiltrating immune cells releasing pro-inflammatory factors and MMPs facilitate the formation of a large lipid core and a thin fibrous cap, which contributes plaque instability.^48^ Our *in vivo* and *ex vivo* studies are the first to demonstrate that circulating hMSCs are capable to adhere at vulnerable endothelium under flow conditions. This suggests that hMSCs are able for subsequent extravasation into plaques, which is in agreement with findings on transplanted MSCs in various animal models of atherosclerosis.^15^

To study the effects of human atherosclerotic plaque on hMSCs, we exposed hMSCs to plaque lysates and investigated changes in cytokine/chemokine transcription. Many of the genes that we found to be up-regulated have important roles in (i) immune cell recruitment, e.g. chemokine C-C motif ligand (CCL)11, CCL18, and chemokine C-X-C motif ligand 2, (ii) pro-inflammation, e.g. IL-1β, CCL13, IL-17, IL-27, and TNF-related apoptosis-inducing ligand (TRAIL), (iii) anti-inflammation e.g. IL-6, IL-13, IL-24 and IL-27, or (iv) cell growth e.g. bone morphogenetic protein 2 (BMP-2), colony-stimulating factor 3 (G-CSF), IL-4, and myostatin. For example, IL-6 is known to polarize monocytes (M0) towards anti-inflammatory IL-10-producing M2 macrophages.^49^ This mechanism, in addition to the direct release of anti-inflammatory factors, might contribute to immunosuppression in plaques as observed upon MSC transplantation in animal models of atherosclerosis.^50^

Furthermore, exposing hMSCs to human plaque lysates augmented the surface expression and/or secretion of MMP-2, MMP-14, and FAP-α as well as TIMP-1, TIMP-2, and TIMP-3. In atherosclerosis, MMPs mainly released from infiltrating immune cells and poorly inhibited by low levels of TIMPs promote mechanical weakening and cap-thinning followed by premature rupture of plaques.^20,21,51^ This detrimental effect of unbalanced MMP activity in plaques might be counteracted by hMSC-derived TIMPs as suggested by studies demonstrating that overexpression of TIMP-1, TIMP-2, and TIMP-3 reduced plaque size.^21^ In addition to their MMP-inhibitory function, TIMPs act as signalling molecules with diverse cytokine-like activities regulating cell proliferation and apoptosis under normal and pathophysiological conditions.^18^ Thus, TIMP-1, TIMP-2, and TIMP-3 secreted from hMSCs in plaques may be athero-protective by inhibiting MMP activity and dampening inflammation.^15^ This is in line with previous work indicating that MSCs transplanted in an animal model of atherosclerosis stabilize vulnerable plaques by reducing MMP expression, decreasing apoptosis, and diminishing levels of inflammatory cytokines.^50^ FAP-α is a cell surface protease mainly expressed on mesenchymal cells and associated with remodelling of extracellular matrix, angiogenesis, and immunosuppression.^52^ The role and function of FAP-α in plaque-exposed hMSCs remains to be elucidated.

Remarkably, our studies provide evidence that human plaque components elicit differentiation of hMSCs into smooth muscle cell-like cells. Interestingly, vulnerable plaques prone to rupture are characterized by only few smooth muscle cells in a thin cap.^53^ Thus, myogenic differentiation of hMSCs in plaque environment might confer a stabilizing effect in vulnerable plaques. In fact, transplanted MSCs were shown to stabilize vulnerable plaques in an animal model of atherosclerosis by strengthening the fibrous cap.^50^ Consistently, we found that human plaque lysates up-regulated endogenous levels of miR-335 in hMSCs, a miRNA shown to promote overall plaque stability.^54^ Because, we demonstrated LL-37 to augment let-7f in hMSCs we expected a similar effect by lysates from plaque known to contain LL-37.^9^ Interestingly, let-7f levels in hMSCs did not change significantly when incubated with human plaque lysates. This observation can be explained by the fact that plaque comprises a plethora of biologically active molecules including inflammatory cytokines, chemokines, as well as proteases and lipoproteins.^4^ These factors are likely to influence various regulatory pathways in hMSCs that may counterbalance the stimulatory effect by plaque-associated LL-37 on let-7f expression in these cells. Overall, let-7f seems to be important in hMSC migration to plaques but is probably less relevant for cell function upon their arrival.

Our study suggests the therapeutic potential of let-7f-overexpressing hMSCs in stabilizing atherosclerotic plaques, nonetheless, some aspects require further investigations. In particular, we did not provide evidence of lower incidence of cardio- and cerebrovascular events upon hMSCs treatment. Unfortunately, plaque rupture and atherothrombosis are rarely observed in murine model of atherosclerosis thus making them unsuitable for studying plaque stability. Although various approaches to favour plaque destabilization in mice have been proposed, none of them fully resemble the human pathophysiology.^51^ Studies on large animal models may represent a future alternative to close this gap before possible progress to clinical trials. Moreover, bioavailability of exogenous hMSCs is reduced upon systemic intravenous injection, thus requiring additional routes of administration for therapeutic applications, e.g. intra-arterial injection during percutaneous coronary intervention or use of hMSC-loaded stents. Finally, although we clearly show the potential of atherosclerosis-relevant mediators to influence let-7f expression and phenotypical changes of hMSCs, the detailed molecular mechanisms will require further scrutiny in transgenic models.

In summary, our findings demonstrate hMSC tropism towards atheromatous tissues that evoke a potentially athero-protective signature by release of paracrine factors and maturation into plaque-stabilizing cells. Clearly, further studies are required to understand the complex roles of hMSCs in atherosclerosis in order to advance the development of innovative approaches using hMSCs in cardiovascular disease therapy.^15^

## Supplementary material

Supplementary material is available at *Cardiovascular Research* online.

## Authors’ contributions

V.E., R.T.A.M., O.S., and C.R. designed research; V.E., R.T.A.M., S.W., and O.S. performed research; V.E., D.S., S.K., and C.R. analysed data; R.M. and O.S. provided mouse model and analysis; R.B. and W.S. provided and processed human plaque tissue specimens; C.R., A.B., and C.W. provided funding, discussion, and supervision; and C.R. prepared the manuscript with input from all co-authors.

## Supplementary Material

cvac022_Supplementary_Data

## Data Availability

Data available on request. Translational perspective Human mesenchymal stem cells (hMSCs) represent a promising therapeutic approach in various pathophysiological processes associated with inflammation including atherosclerosis. The current knowledge about the mechanisms of hMSC tropism towards human atherosclerotic plaques and their beneficial effects at the site is poor. Bridging this gap is essential for clinical application of hMSCs. Our work provides insight into the contribution of microRNA let-7f in hMSC recruitment to atheroprone areas, where hMSCs display athero-protective potential by releasing immunomodulatory factors and differentiating towards plaque-stabilizing cells. Our findings highlight circulating hMSCs as a possible therapeutic strategy for the stabilization of atherosclerotic plaques.
