# Supplementary material for: Properties and fate of human mesenchymal stem cells upon miRNA let-7f-promoted recruitment to atherosclerotic plaques
Source: Cardiovasc Res. 2022 Mar 3;119(1):155–66. doi: 10.1093/cvr/cvac022 (PMC10022860; doi:10.1093/cvr/cvac022)
Supplement: cvac022_Supplementary_Data [file cvac022_supplementary_data.zip › Supplementary_data.docx]

Cardiovascular Research

**Properties and fate of human mesenchymal stem cells upon
miRNA let-7f-promoted recruitment to atherosclerotic plaques**

**Short title:** hMSC tropism and fate in atherosclerotic plaques

Virginia Egea^1^*, Remco Theodorus Adrianus Megens^1,2,3^, Donato Santovito^1,2,4^, Sarawuth Wantha^1^, Richard Brandl^5^, Wolfgang Siess^1^, Sajjad Khani^1^, Oliver Soehnlein^1,6,7^, Alexander Bartelt^1,2,8,9^, Christian Weber^1,10,11^, Christian Ries^1^*.

Supplementary tables, figures, videos, and unedited Western blots

# Supplementary Tables

# Supplementary Figures

**Fig. S1. Arterial tissue isolation, processing, and incubation with hMSCs.**

**A-C)** Pieces of the atherosclerotic plaque core area or the nearby normal-appearing intima in the same vessel specimen were removed from tissue that had been preserved from patients who underwent endarterectomy. Scale: cm. **D)** Tissue homogenates (100 mg wet weight each) from 6 patients were processed to obtain extracts that were applied for incubation with hMSCs as described in detail in the Material and Methods section of the main manuscript.

# Supplementary Videos

**Supplementary video 1:** Carotid arteries were explanted from *Apoe*^–/–^ mice fed with high fat diet. A 1:1 mixture of red and green labeled hMSCs transfected with let-7f or non-specific control were perfused through the mounted vessel and analyzed by 3D image acquisition using two-photon laser scanning microscopy as described in detail in Material and Methods.

# Unedited western blots
