# Supplementary material for: Properties and fate of human mesenchymal stem cells upon miRNA let-7f-promoted recruitment to atherosclerotic plaques
Source: Cardiovasc Res. 2022 Mar 3;119(1):155–66. doi: 10.1093/cvr/cvac022 (PMC10022860; doi:10.1093/cvr/cvac022)
Supplement: cvac022_Supplementary_Data [file cvac022_supplementary_data.zip › Supplementary_Tables.docx]

# Supplementary Tables

**Table S1.** Sequence of let-7f miRNA mimic, control, and qRT-PCR primer sets.

| **Hsa transcript** | **Supplier and Catalog number** |
| --- | --- |
| Let-7f-3p mimic | Qiagen miScript miRNA mimic cat. no. MSY0000067 |
| Control oligonucleotide | Qiagen AllStars Negative Control siRNA cat. no. 1027280 |
| Let-7f-3p | Qiagen miScript cat. no. MS00008274 |
| MiR-335-5p | Qiagen miScript cat. no. MS00003976 |
| Snord61 | Qiagen miScript cat. no. MS00033705 |
| Snord72 | Qiagen miScript cat. no. MS00033719 |
| U2 | Qiagen QuantiTect Primer Assay cat. no. QT00097594 |
| GAPDH | Qiagen QuantiTect Primer Assay cat. no. QT02504278 |
| FPR2 | Qiagen QuantiTect Primer Assay cat. no QT00204295 |
| PARP delta | Search-LC cat. no. n/a |
| Osteocalcin | Search-LC cat. no. n/a |
| Decorin | Search-LC cat. no. n/a |
| Akaline phosphatase | Search-LC cat. no. n/a |
| αSMA | Search-LC cat. no. n/a |
| Caldesmon | Search-LC cat. no. n/a |
| Interleukin 6 | Search-LC cat. no. 487553 |
| TIMP-1 | Search-LC cat. no. 487560 |
| TIMP-2 | Search-LC cat. no. n/a |
| TIMP-3 | Search-LC cat. no. 487584 |
| MMP-2 | Search-LC cat. no. 487651 |
| MMP-14 | Search-LC cat. no. n/a |
| FAPα | Search-LC cat. no. n/a |

Hsa, Homo sapiens. Cat. no. n/a, catalogue number not available.

**Table S2.** List of antibodies.

| **Antigen** | **Supplier** | **Clone /**  **Cat. no.** | **Concentration** | |
| --- | --- | --- | --- | --- |
|  |  |  | **WB/FACS** | **IC** |
| FPR2 | R&D systems | 304405 | 1:100 | - |
| αSMA | Abcam | AB15734 | 1 µg/ml | 1:250 |
| Caldesmon | Abcam | AB68878 | 1 µg/ml | 1:250 |
| Interleukin 6 | R&D Systems | AB-206-NA | 1:1000 | ~~-~~ |
| TIMP-1 | Abcam | AB61224 | 1:1000 | ~~-~~ |
| TIMP-2 | Abcam | AB1828 | 1:1000 | ~~-~~ |
| TIMP-3 | AssayBioTech | C0349 | 1:1000 | ~~-~~ |
| MMP-2 | Calbiochem | 75-7F7 | 1:1000 | ~~-~~ |
| MMP-2 | Chemicon | MAB3308 | 1:1000 | ~~-~~ |
| MMP-14 | Chemicon | AB815 | 1:1000 | ~~-~~ |
| FAPα | R&D Systems | AF3715 | 1:1000 | ~~-~~ |
| LIN28A/B | Cell Signaling | 4196S | 1:1000 |  |
| β-actin | Abcam | ab8227 | 1:100000 | - |
| Goat anti-rabbit IgG (Star635P) | Abberior | ST635P-1002 | ~~-~~ | 1:500 |
| Horse anti-mouse IgG (HRP) | Cell Signaling | 7076 | 1:2000 | ~~-~~ |
| Goat anti-rabbit IgG (HRP) | Cell Signaling | 7074 | 1:2000 | ~~-~~ |
| Donkey anti-sheep IgG (HRP) | TFS | A16041 | 1:2000 | ~~-~~ |
| Donkey anti-goat IgG (HRP) | Abcam | AB97110 | 1:2000 | ~~-~~ |

WB, Western blot; IC, immunocytochemistry; FACS, flow cytometry
TFS, Thermo Fisher Scientific; HRP, horse-raddish-peroxidase-linked
