# Supplementary figures and images for: Properties and fate of human mesenchymal stem cells upon miRNA let-7f-promoted recruitment to atherosclerotic plaques
Source: Cardiovasc Res. 2022 Mar 3;119(1):155–66. doi: 10.1093/cvr/cvac022 (PMC10022860; doi:10.1093/cvr/cvac022)

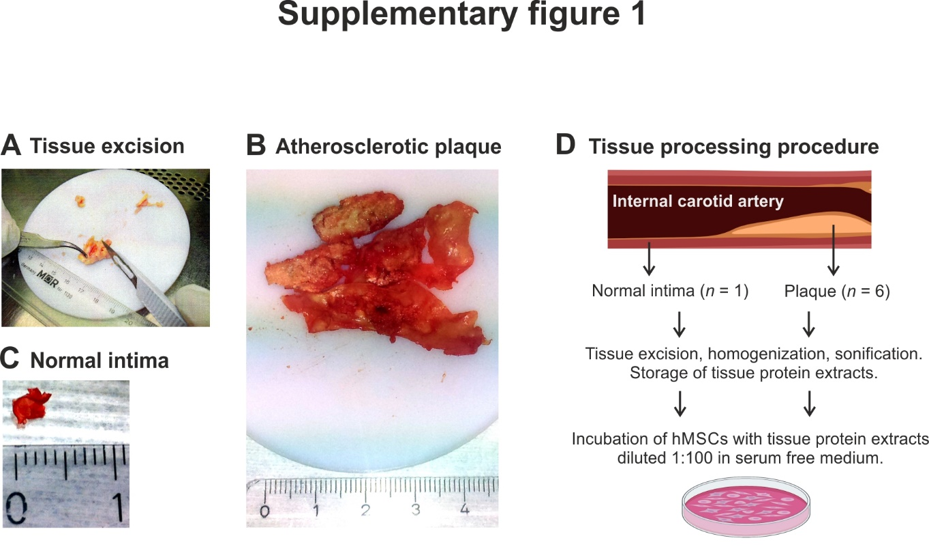

Supplement: cvac022_Supplementary_Data [file cvac022_supplementary_data.zip › Fig_S1.tif]
